# Supplementary material for: Personalized Biopsy Schedules Using an Interval‐Censored Cause‐Specific Joint Model
Source: Stat Med. 2025 May 26;44(10-12):e70134. doi: 10.1002/sim.70134 (PMC12104802; doi:10.1002/sim.70134)
Supplement: Supplementary file 1 — Data S1. Additional supporting information may be found in the online version of the article at the publisher's website. [file SIM-44-0-s001.pdf]

Supporting material - Personalized Biopsy Schedules Using an Interval-censored Cause-specific Joint Model

Zhenwei Yang[1,2], Dimitris Rizopoulos[1,2], Eveline A.M. Heijnsdijk[3], Lisa F. Newcomb[4], and Nicole S. Erler[1,2,5]

[1]Department of Biostatistics, Erasmus Medical Center Rotterdam
[2]Department of Epidemiology, Erasmus Medical Center Rotterdam
[3]Department of Public Health, Erasmus Medical Center Rotterdam
[4]Fred Hutchinson Cancer Center, Cancer Prevention Program, Public Health Sciences, Seattle, Washington
[5]Julius Center for Health Sciences and Primary Care, University Medical Center Utrecht, Utrecht University, Utrecht

October 3, 2024

Web Appendix 1 Data

Web Appendix 1.1 PASS Data

Table S1 summarizes the relevant subset of the Canary PASS data.

Table S1: Summary table for the Canary PASS Data.

**Item**	**Value**
Number of subjects	833
Observation time until progression/treatment (years)*	4.35 (2.82-6.18)
Baseline PSA density‡ (ng/ml^2)†	0.12 (0.10)
Age at start of AS (years)*	62 (57-67)
Total number of PSA measurements	8262
Number of PSA measurements per patient*	9 (5-14)
PSA level (ng/ml)†	5.10 (3.84)
Number of positive cores per patient*	3 (2-4)
core ratio (%)*	8.33 (0.00-16.67)
Number of biopsies per patient*	2 (2-3)

* median is shown followed by the interval between 25% quantile and 75% quantile;
† mean is shown with standard deviation in the parentheses;
‡: PSA density equals to PSA level (ng/ml) divided by prostate volume (ml).

Web Appendix 1.2 Time-to-event Outcomes

The Aalen–Johansen estrimator of the two events' risks in the PASS data is visualized in Figure S1.

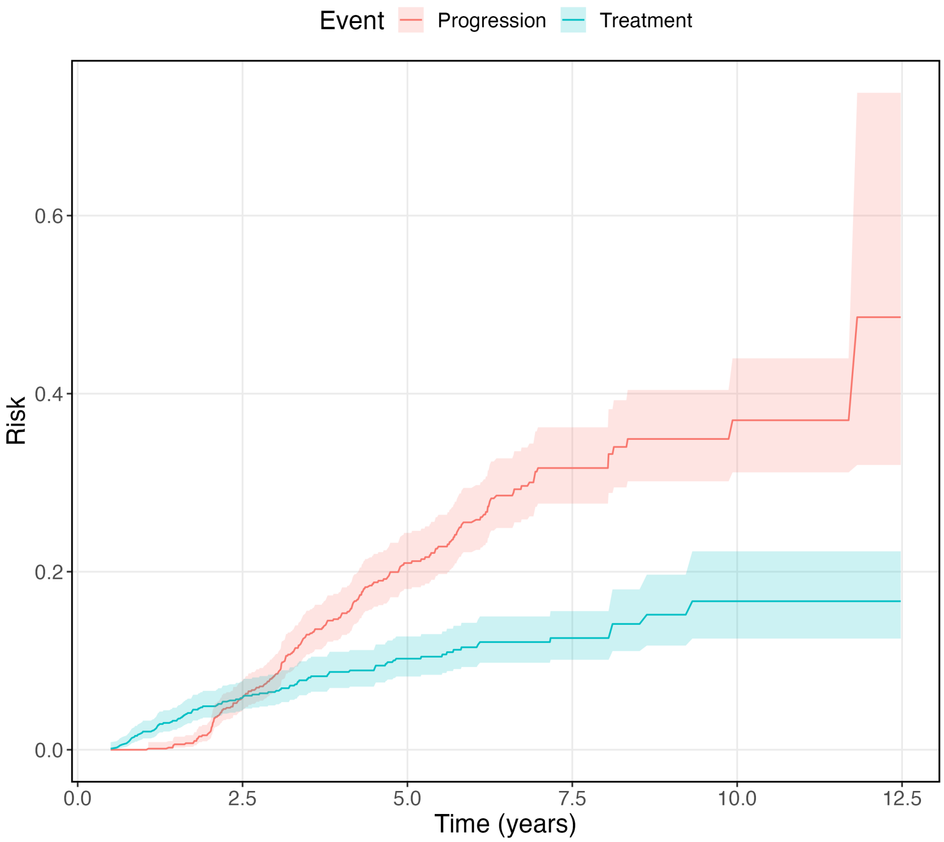

Figure S1: The Aalen-Johansen estimator of progression- and treatment-specific risk in the PASS data.

Web Appendix 1.3 Longitudinal Outcomes

In the Canary PASS data, two longitudinal outcome are available, namely, PSA levels and proportion of cores obtained by a biopsy that contain cancer cells (core ratio). In Figure S2, the development of the longitudinal outcomes is displayed for 20 randomly selected patients. The trajectories show non-linear evolutions over time, and vary greatly between patients, which needs to be accommodated in the longitudinal component of the ICJM.

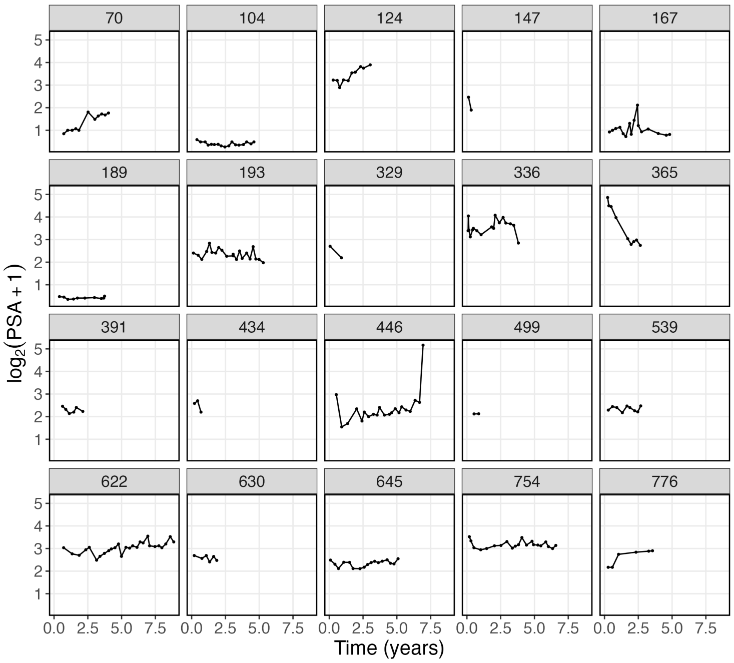

(a) PSA levels

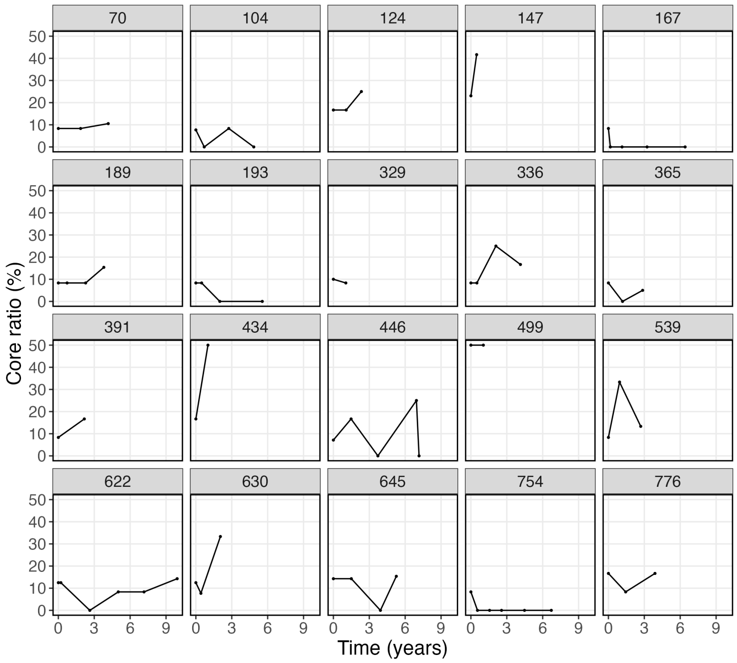

(b) Core ratios

Figure S2: Observed trajectories of two longitudinal outcomes for 20 randomly selected subjects.

Web Appendix 2 Interval-censored Cause-specific Joint Models (ICJM) for Longitudinal and Time-to-event Data

In the specification of the ICJM, we use a flexible semi-parametric specification of the baseline hazard using penalized B-splines,

$$\log h_0^{(k)}(t) = \gamma_{k,h_0,0} + \sum_{a=1}^{A} \gamma_{k,h_0,a} \mathcal{G}_a(t, \boldsymbol{\xi}),$$

where $\mathcal{G}_a(t, \boldsymbol{\xi})$ is the a-th basis function of a B-splines with knots $\xi_1, \ldots, \xi_A$. The number of knots was chosen to be 11. The penalized coefficients for the basis function $\boldsymbol{\gamma}_{k,h_0}$ have the following priors,

$$p(\boldsymbol{\gamma}_{k,h_0} \mid \tau_{k,h_0}) \propto \tau_{k,h_0}^{\rho(\boldsymbol{M})/2} \exp\left(-\frac{\tau_{k,h_0}}{2} \boldsymbol{\gamma}_{k,h_0}^\top \boldsymbol{M} \boldsymbol{\gamma}_{k,h_0}\right),$$

with

$$\tau_{k,h_0} \sim \text{Gamma}(5, 0.5),$$

where τ_{k,h_0} is the smoothing parameter; $\boldsymbol{M} = \Delta_r^\top \Delta_r + 10^{-6} I$, Δ_r is the r-th difference penalty matrix and $\rho(\boldsymbol{M})$ denotes the rank of $\boldsymbol{M}$.

Web Appendix 3 Simulation Study

Web Appendix 3.1 Simulation Setting

To evaluate the performance of our proposed methodology, we simulated data based on the parameters from the ICJM fitted on the Canary PASS study (ICJM 1, see Section 5). The patients in the training sets are supposed to take biopsies in months 12, 24 and afterwards biennially and PSA measurements every three months, with small variations.

This model had the following structure:

$$\begin{aligned}
\log_2(\text{PSA}_i + 1)(t) &= m_{\text{PSA},i}(t) + \epsilon_i(t), \\
m_{\text{PSA},i}(t) &= \beta_0 + u_{0i} + \sum_{p=1}^{3} (\beta_p + u_{pi}) \mathcal{C}_i^{(p)}(t) + \beta_4(\text{Age}_i - 62), \\
h_i^{(k)}\left\{t \mid \boldsymbol{\mathcal{M}}_{\text{PSA},i}(t)\right\} &= h_0^{(k)}(t) \exp\Big[\gamma_k \text{density}_i + f\left\{\boldsymbol{\mathcal{M}}_{\text{PSA},i}(t), \boldsymbol{\alpha}_k\right\}\Big],
\end{aligned}$$

where $\mathcal{C}(t)$is the design matrix for the natural cubic splines (with three degrees of freedom) for time t; Age_i and density_i refer to the patient's age and PSA density at the start of active surveillance, respectively. Baseline Age was centered by subtracting the median age (62 years) for computational reasons. Both the expected value of PSA and the change in expected PSA over the previous year (where extrapolation was conducted for time points earlier than year one) were included as covariates in the time-to-event component, i.e.,

$$f\left\{\boldsymbol{\mathcal{M}}_{\text{PSA},i}(t), \boldsymbol{\alpha}_k\right\} = \alpha_{1k,\text{PSA}} m_{\text{PSA},i}(t) + \alpha_{2k,\text{PSA}} \Big\{m_{\text{PSA},i}(t) - m_{\text{PSA},i}(t-1)\Big\}.$$

The residuals of the longitudinal component were assumed to follow a Student's t distribution with three degrees of freedom [1],

$$\epsilon_i(t) \sim t(\frac{1}{\tau_\epsilon}, 3),$$

with

$$\tau_\epsilon \sim \text{Gamma}(0.01, 0.01).$$

The prior distributions for the regression coefficients were specified as vague normal distributions,

$$\begin{aligned} \beta &\sim \mathcal{N}(0, 100), \\ \gamma_k &\sim \mathcal{N}(0, 100), \\ \alpha_{1k,\text{PSA}}, \alpha_{2k,\text{PSA}} &\sim \mathcal{N}(0, 100), \end{aligned}$$

and the variance-covariance matrix of the random effects, $\mathbf{\Omega}$, to follow an inverse-Wishart distribution,

$$\mathbf{\Omega} \sim \mathcal{IW}(n_u + 1, \frac{4}{\tau_u}),$$

with

$$\tau_u \sim \text{Gamma}(0.5, 0.01),$$

where n_u is the number of coefficients for random effects.

The model was implemented in JAGS [2] and run for 10000 iterations, using a thinning interval of 10, in each of three MCMC chains.

The resulting posterior means used for simulation were

$$
\begin{aligned}
\boldsymbol{\beta} &= [2.34, 0.28, 0.61, 0.95, 0.02]^\top, \\
\boldsymbol{\Omega} &= \begin{bmatrix} 0.48 & -0.04 & -0.07 & 0.02 \\ -0.04 & 0.77 & 0.46 & -0.04 \\ -0.07 & 0.46 & 1.37 & 1.36 \\ 0.02 & -0.04 & 1.36 & 2.54 \end{bmatrix}, \\
\tau_\epsilon &= 47.40, \\
\boldsymbol{\gamma}_{h_0} &= \begin{bmatrix} -6.78 & -5.76 \\ -4.72 & -4.99 \\ -2.84 & -4.43 \\ -1.65 & -4.26 \\ -1.54 & -4.36 \\ -1.79 & -4.47 \\ -1.85 & -4.60 \\ -1.75 & -4.69 \\ -1.85 & -4.78 \\ -2.04 & -4.92 \\ -2.18 & -5.08 \\ -2.32 & -5.21 \end{bmatrix}, \\
\boldsymbol{\gamma} &= [0.50, 0.23], \\
\boldsymbol{\alpha} &= \begin{bmatrix} 0.13 & 0.42 \\ 3.01 & 2.62 \end{bmatrix}.
\end{aligned}
$$

The resulting simulated data matched the observed data well with regard to the rates of cancer progression, early treatment initiation and censoring (Table S2).

Table S2: Summary of event proportions in the simulated training datasets compared to the observed data.

**Events**	**Simulated data**† (%)	**Observed data** (%)
Cancer progression	28.29	21.97
Treatment	7.92	10.44
Censoring	63.79	67.59

†: the average proportions overall training sets are presented.

Web Appendix 3.2 Evaluation of the ICJM

Since the quality of the personalized schedules relies on good predictive accuracy, we investigated the prediction error of the ICJM.

We predicted the 2-year cancer progression risk at different starting points (baseline, year 1, 2, 3, 4 and 6) assuming patients just experienced the biopsies, based on (8) from the manuscript, for all patients in the test sets based on the ICJMs fitted on the corresponding training sets and calculated the prediction error (i.e., the difference between the true and predicted risk) in R [3].

Figure S3 visualizes these results; it shows that the ICJM slightly overestimates the 2-year progression risk for later years. Further investigation revealed that this likely stems

from the overestimation of the baseline hazard, as shown in Figure S4. The placement of the knots in the spline specification of the baseline hazard is based on the quantiles of the event times in the training data. Since most events occur between years 3 and 4, there is little information to guide the spline fit in the period after year 5.

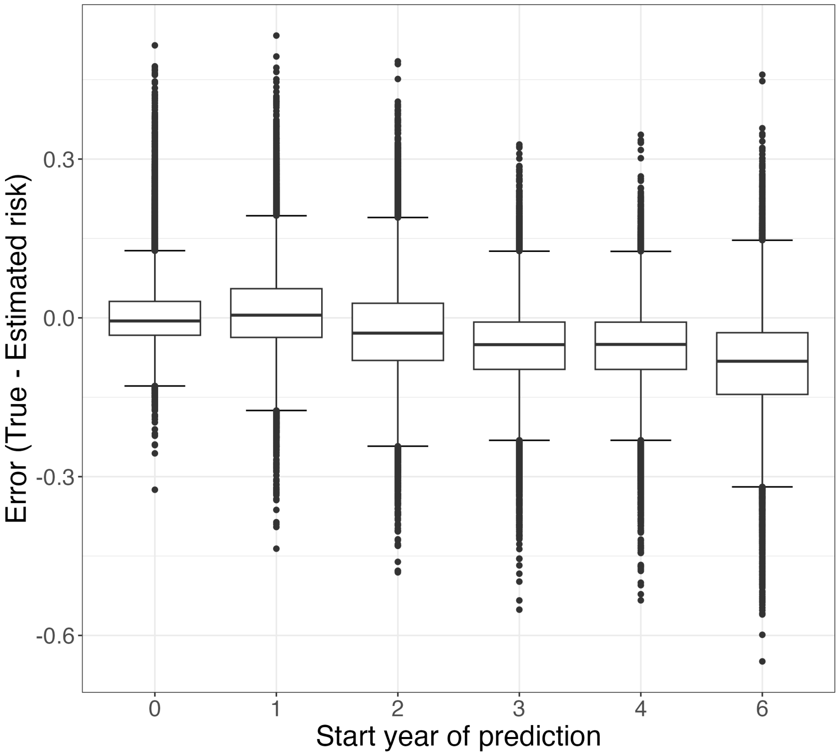

Figure S3: Two-year risk prediction performance evaluation for 200 test sets (True risks are calculated based on the true parameters including the patient-specific random effects used to generate the simulated data).

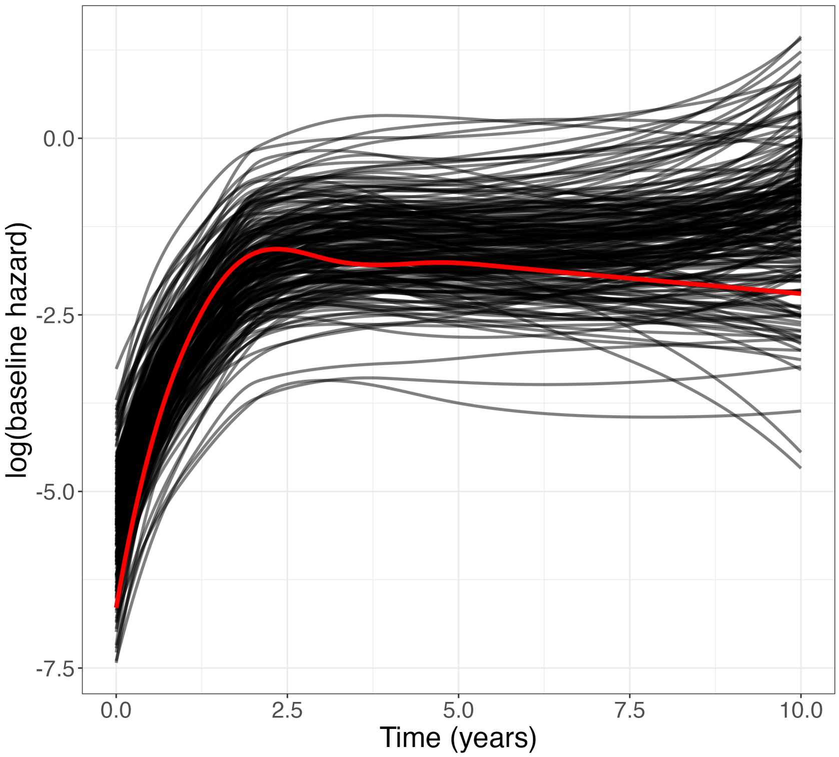

Figure S4: Visualization of the baseline hazards over time (red curve: true baseline hazard ; black curves: estimated baseline hazards).

Web Appendix 4 ICJM for Data Analysis

The model specification of our primary model, ICJM 1, is detailed in Web Appendix 3.1.

Web Appendix 4.1 Model Specification of ICJM 2

To investigate the role of the core ratio as a potential predictor for cancer progression and to demonstrate how to incorporate additional biomarkers in the ICJM, we extended ICJM with a binomial mixed model for the core ratio. In this model, we assumed a quadratic evolution over time. The random effects were modelled jointly with the random effects in the model for PSA, i.e.,

$$\log_2(\text{PSA}_i + 1)(t) = m_{\text{PSA},i}(t) + \epsilon_i(t),$$

$$m_{\text{PSA},i}(t) = \beta_0 + u_{0i} + \sum_{p}^{3}(\beta_p + u_{pi})\mathcal{C}_i^{(p)}(t) + \beta_4(\text{Age}_i - 62),$$

$$\text{logit}\left[E\{\text{core ratio}_i(t)\}\right] = m_{2i}(t),$$

$$m_{2i}(t) = \beta_5 + u_{4i} + (\beta_6 + u_{5i})t + (\beta_7 + u_{6i})t^2,$$

where the random effects from the two longitudinal outcomes $\boldsymbol{u}_i = (u_{1i}, \ldots, u_{6i})^\top$ are modeled jointly using a multivariate normal distribution, $\boldsymbol{u}_i \sim \mathcal{N}(0, \boldsymbol{\Omega})$.

The survival component of the ICJM was extended to also include the estimated trajectory of the core-ratio, $\boldsymbol{\mathcal{M}}_{\text{CR},i}(t)$,

$$h_i^{(k)}\left\{t \mid \boldsymbol{\mathcal{M}}_{\text{PSA},i}(t), \boldsymbol{\mathcal{M}}_{\text{CR},i}(t)\right\} = h_0^{(k)}(t) \exp\Big[\gamma_k \text{density}_i + f\left\{\boldsymbol{\mathcal{M}}_{\text{PSA},i}(t), \boldsymbol{\mathcal{M}}_{\text{CR},i}(t), \boldsymbol{\alpha}_k\right\}\Big],$$

where $\boldsymbol{\alpha}_k = [\alpha_{1k,\text{PSA}}, \alpha_{2k,\text{PSA}}, \alpha_{1k,\text{CR}}]$ and $f\left\{\boldsymbol{\mathcal{M}}_{\text{PSA},i}(t), \boldsymbol{\mathcal{M}}_{\text{CR},i}(t), \boldsymbol{\alpha}_k\right\}$ now also included the expected value of the core ratio,

$$\begin{aligned} f\left\{\boldsymbol{\mathcal{M}}_{\text{PSA},i}(t), \boldsymbol{\mathcal{M}}_{\text{CR},i}(t), \boldsymbol{\alpha}_k\right\} = {} & \alpha_{1k,\text{PSA}} m_{\text{PSA},i}(t) + \alpha_{2k,\text{PSA}}\Big\{m_{\text{PSA},i}(t) - m_{\text{PSA},i}(t-1)\Big\} \\ & + \alpha_{1k,\text{CR}} m_{\text{CR},i}(t). \end{aligned}$$

ICJM 2 was fitted in JAGS, using 10000 iterations, using a thinning interval of 10, in each of three MCMC chains.

Web Appendix 4.2 Goodness of fit

The goodness of fit the ICJM depends on the fit of individual expected trajectories of the biomarkers. Therefore, we examined the fitted trajectories of PSA levels and core ratios for the 20 selected subjects in Figure S2. The results are visualized in Figure S5. It is shown that the ICJM is able to generally capture the non-linear trends for the biomarkers of each individual patient.

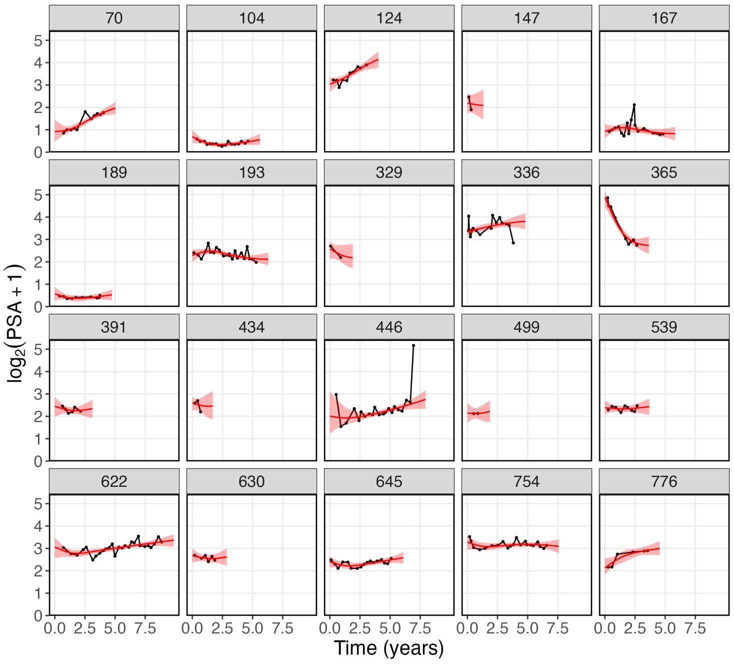

(a) PSA levels

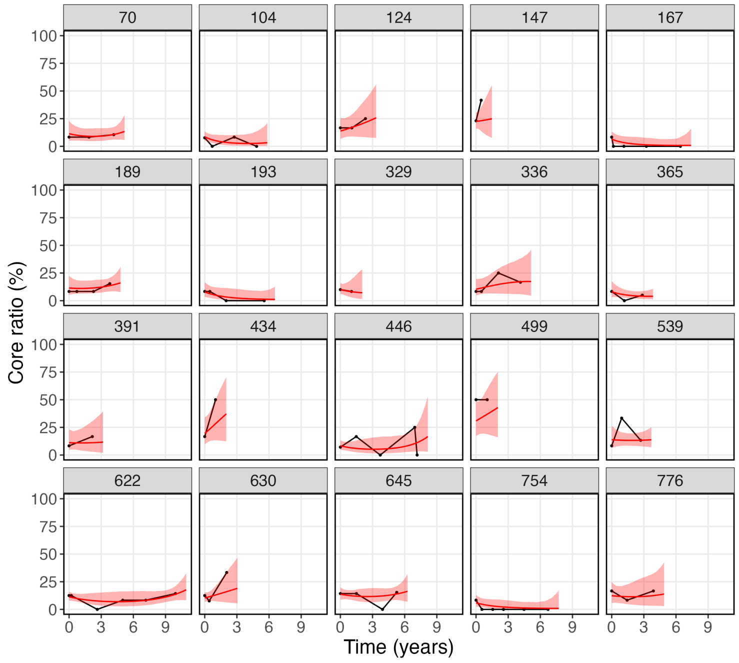

(b) Core ratios

Figure S5: Fitted trajectories of two longitudinal outcomes for 20 randomly selected subjects.

Web Appendix 4.3 Results

The results from ICJM 1 and ICJM 2 are presented in Table S3. Since the coefficients pertaining to the natural cubic splines used to model the non-linearity of the trajectories of the PSA levels do not have a direct and clinically meaningful interpretation and to facilitate the interpretation, we present effect plots of the estimated PSA level trajectory and core ratio trajectory for patients with the median age of 62 years in Figure S6. Both outcomes remained stable at the beginning and increased with time.

Table S3: Summary of the model parameter estimates in ICJM 1 and ICJM 2.

**Parameters**	**ICJM 1 (PSA)**		**ICJM 2 (PSA + core ratio)**	
	**Estimate**	**95% CI**	**Estimate**	**95% CI**
**Longitudinal component - PSA**				
Intercept	2.35	[2.30, 2.40]	2.35	[2.30, 2.40]
Time 1†	0.28	[0.19, 0.37]	0.29	[0.19, 0.37]
Time 2†	0.59	[0.41, 0.74]	0.64	[0.50, 0.79]
Time 3†	0.94	[0.62, 1.21]	1.04	[0.80, 1.32]
Age	0.02	[0.01, 0.02]	0.02	[0.01, 0.02]
**Longitudinal component - core ratio**				
Intercept	-	-	-2.09	[-2.14, -2.03]
Time	-	-	-0.20	[-0.28, -0.13]
Time^2	-	-	0.04	[0.03, 0.05]
**Progression-specific survival component**				
log(PSA density)	0.51	[0.23, 0.77]	0.30	[-0.03, 0.58]
$\log_2(\text{PSA}+1)$ value	0.13	[-0.11, 0.36]	0.26	[0.02, 0.50]
$\log_2(\text{PSA}+1)$ yearly change	2.92	[1.75, 4.10]	1.65	[0.30, 2.98]
logit[E(core ratio)] value	-	-	1.10	[0.91, 1.29]
**Treatment-specific survival component**				
log(PSA density)	0.22	[-0.18, 0.63]	-0.25	[-0.79, 0.26]
$\log_2(\text{PSA}+1)$ value	0.42	[0.08, 0.74]	0.54	[0.13, 0.93]
$\log_2(\text{PSA}+1)$ yearly change	2.21	[0.16, 4.21]	0.51	[-1.69, 2.82]
logit[E(core ratio)] value	-	-	1.54	[1.20, 1.89]

† Time variable is specified using natural cubic splines with 3 degrees of freedom.

CI: credible interval

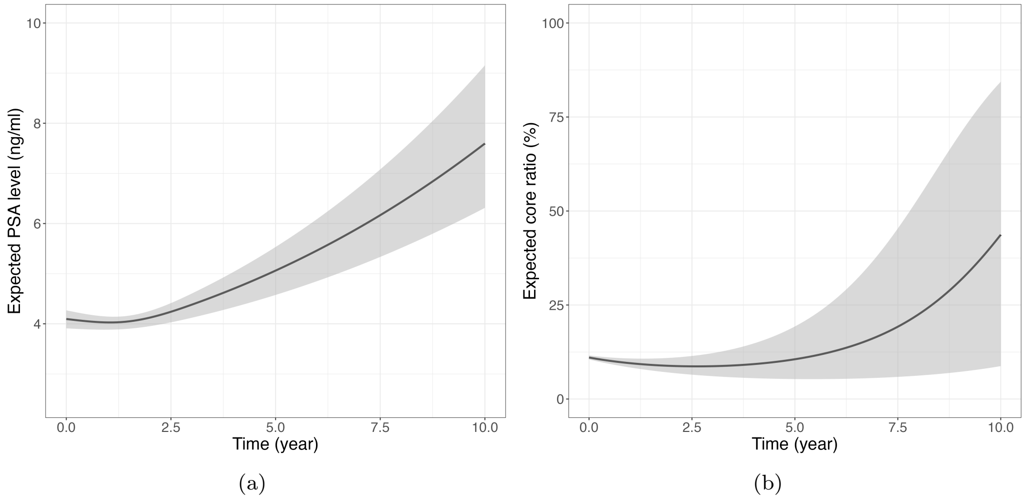

Figure S6: Effect of time in the longitudinal modeling of (a) the PSA level and (b) the core ratio (ICJM 2).

The estimated effect of the core ratio is visualized in Figure S7. A one-fold increase of the core ratio to 30% raised the progression-specific risk by a factor of 2.66 times while halving the core ratio lowered the risk by 58%.

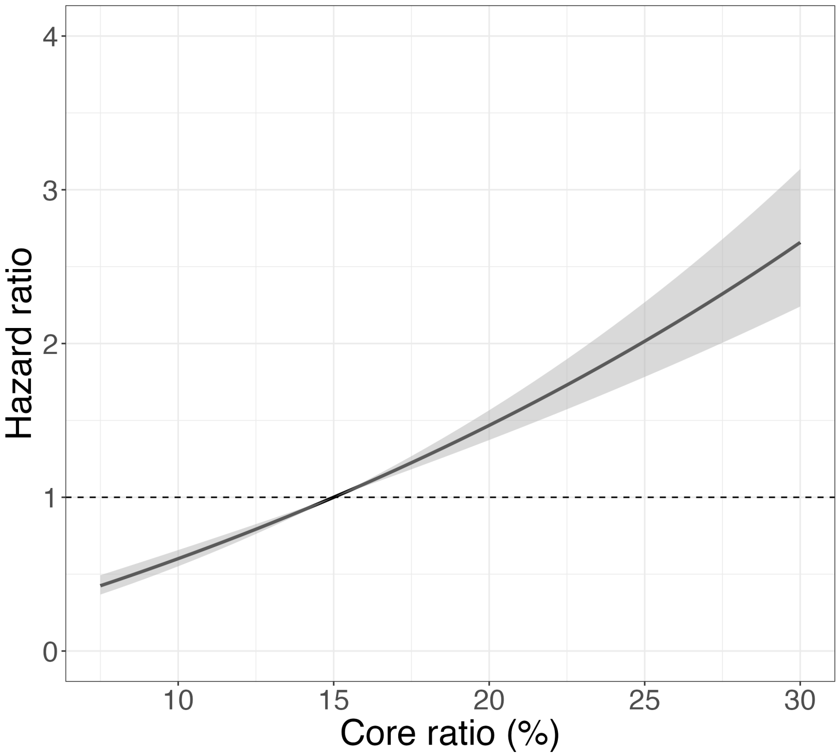

Figure S7: Effect of the core ratio value (contrast to a core ratio of 15%) on the risk of progression, considering the baseline PSA density, the current PSA level and PSA magnitude of change over the past year remain constant.

Web Appendix 4.4 Importance of including the competing risks

To provide an indication of the relevance of treating early treatment initiation as a competing event, we fitted the mis-specified model in which patients were censored at the time of early treatment initiation. The results of this model are presented in Table S3, alongside the results of the corresponding model with competing risk, ICJM 1.

The important coefficients of both models are represented in Table S3. We observed considerable differences between the two models in the association parameters of the PSA value and yearly change, indicating different risk estimates for the two models.

Table S4: Summary of the model parameter estimates in ICJM 1 (with early treatment as a competing risk) and the interval-censored JM (in which the competing event was treated as censoring).

**Parameters**	**Interval-censored JM**		**ICJM 1**	
	**Estimate**	**95% CI**	**Estimate**	**95% CI**
**Longitudinal component - PSA**				
Intercept	2.29	[2.25, 2.34]	2.35	[2.30, 2.40]
Time 1†	0.44	[-0.91, 1.67]	0.28	[0.19, 0.37]
Time 2†	0.74	[0.14, 1.42]	0.59	[0.41, 0.74]
Time 3†	0.77	[-1.14, 2.16]	0.94	[0.62, 1.21]
Age	0.02	[0.01, 0.02]	0.02	[0.01, 0.02]
**(Progression-specific) survival component**				
log(PSA density)	0.52	[0.28, 0.76]	0.51	[0.23, 0.77]
$\log_2(\text{PSA}+1)$ value	0.30	[0.10, 0.50]	0.13	[-0.11, 0.36]
$\log_2(\text{PSA}+1)$ yearly change	0.32	[-0.71, 1.3]	2.92	[1.75, 4.10]

† Time variable is specified using natural cubic splines with 3 degrees of freedom.

CI: credible interval

The estimated log-baseline hazards of both models are visualized in Figure S8. Until year three, the simple standard interval-censored JM underestimates the baseline hazard compared to the ICJM 1.

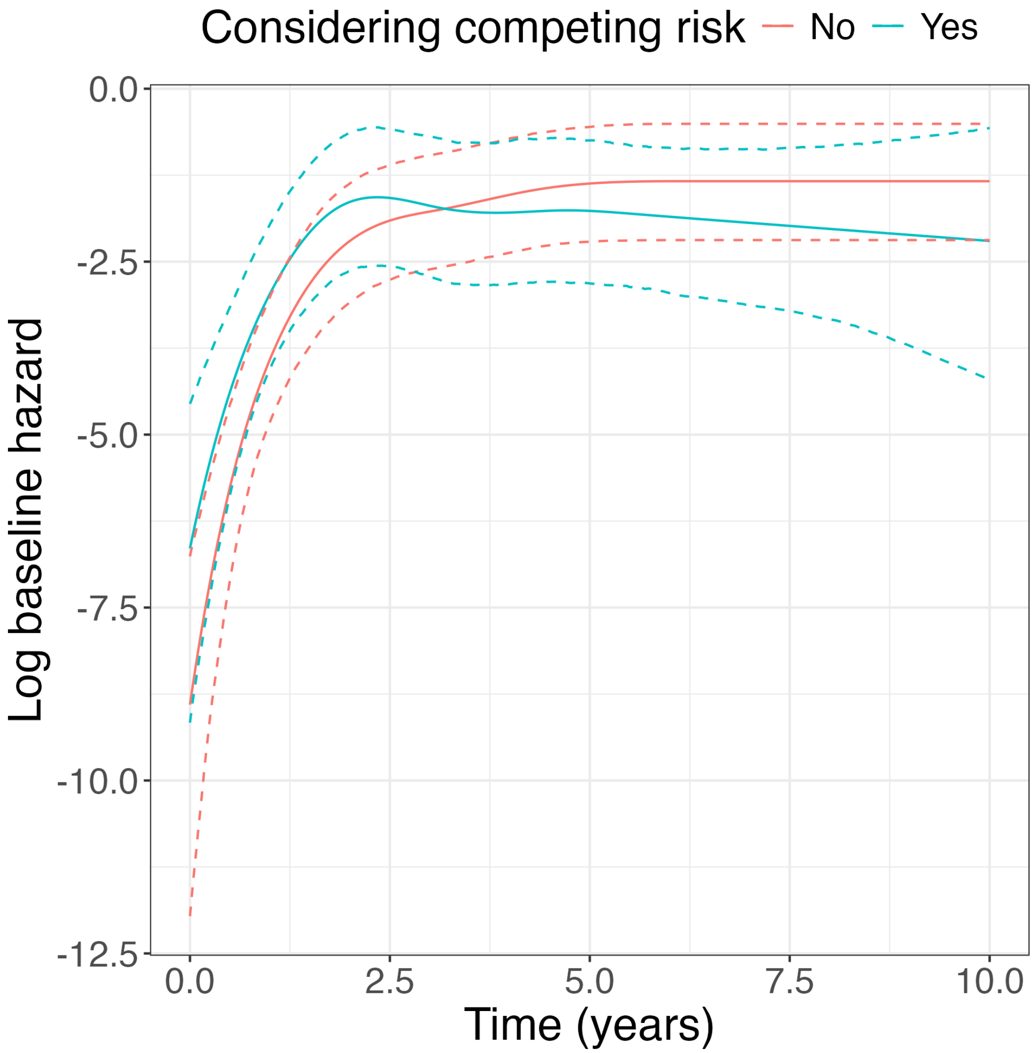

Figure S8: Baseline hazards comparison between including and ignoring the competing risks.

Figure S9 shows the estimated progression-specific cumulative risk for one example patient, conditional on his for PSA measurements available for the first year, for the ICJM and the corresponding model in which patients with the competing event were treated as censored. The plot shows an considerable deviation of the two curves from 4.5 years onwards. The risk differences for this specific patient at different time points between the ICJM and the standard interval-censored JM had a maximum of 5.99%, a minimum of -25.87%, and a median of -0.09%. This difference in the estimated risk indicates that the resulting schedules may considerably differ when the competing event is incorrectly treated as censoring.

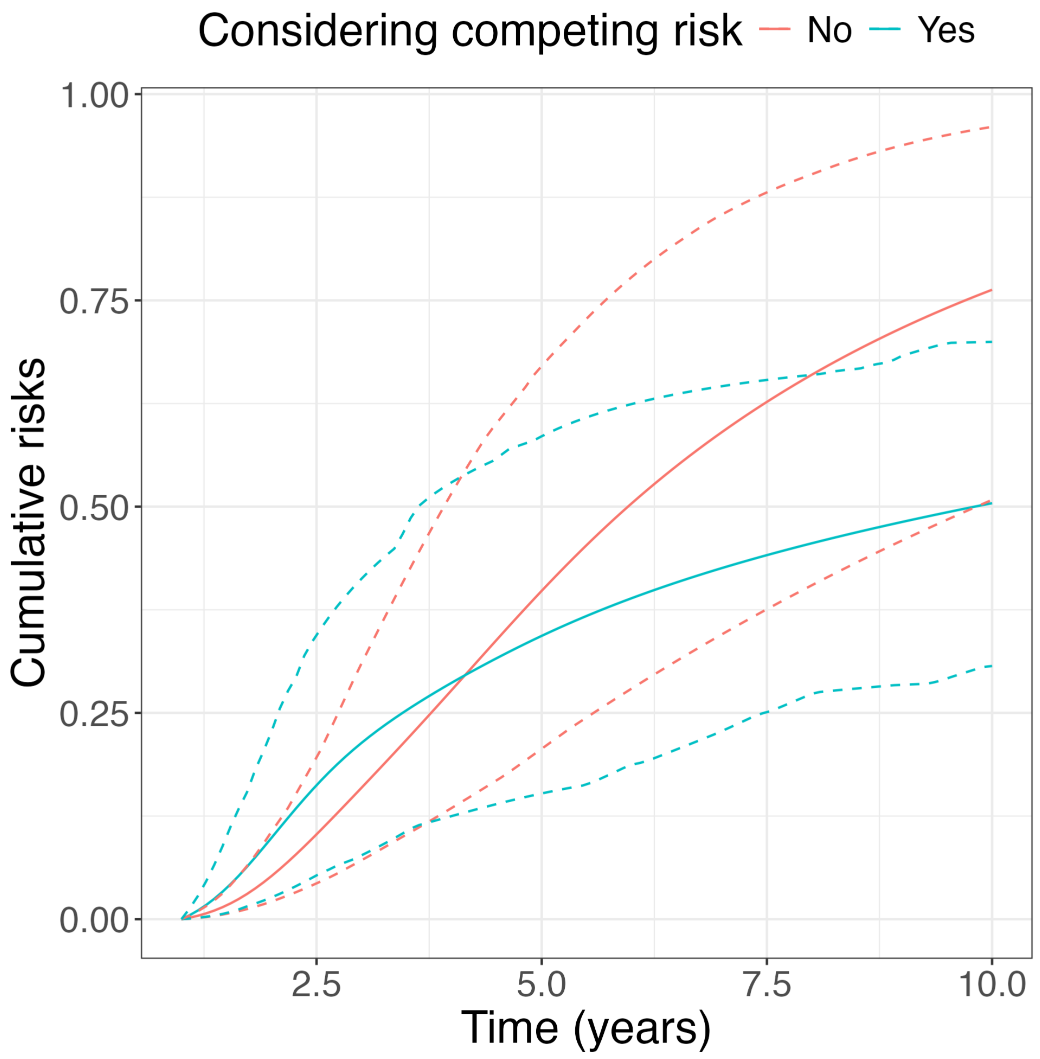

Figure S9: Progression-specific risks (from year one, using the PSA before year one) comparison between including and ignoring the competing risks.

Web Appendix 5 Example of a personalized biopsy scheduling procedure

In this section, we provide an example to illustrate the dynamic personalized biopsy scheduling methodology.

Imagine patient i' started AS two years ago (i.e., the current time is 2, we currently at visit number $v = 4$, i.e., the time of the fourth visit is $t_4 = 2$) and has just had his regular PSA measurement (at year 2). So far, he has had a biopsy when he entered AS (i.e., $t^{(b)} = 0$), and from this biopsy, the core ratio was determined. So we know the history of

the longitudinal outcomes for patient i' up until $t^{(y)} = 2$.

To determine whether a biopsy should be performed at the current visit, we use (3) from the manuscript. Assume the risk threshold ϕ is 0.1 and the calculated progression specific risk $\Pi_{i'}^{(\text{PRG})}\left\{t_4 \mid t^{(b)}, t^{(y)}\right\} = \Pi_{i'}^{(\text{PRG})}\{2 \mid 0, 2\} = 0.08$. Since the risk is between the threshold, no biopsy is performed at this time.

To create a schedule of the expected future biopsy times for patient i', we also predict the risk at the subsequent clinical visits, i.e., times $2.5, 3, 3.5, \ldots$. For predicting the risk at the next (i.e., fifth) visit, at $t_5 = 2.5$, the information on the longitudinal outcomes remains the same since we are still at time 2, and only the time for which we want to estimate the risk has changed. Say, the expected risk $\Pi_{i'}^{(\text{PRG})}\left\{t_5 \mid t^{(b)}, t^{(y)}\right\} = \Pi_{i'}^{(\text{PRG})}\{2.5 \mid 0, 2\} = 0.11$ and, thus, larger than the threshold $\phi = 0.1$. A biopsy will be scheduled for the visit at 2.5 years.

For calculating the expected risk at the subsequent visit times, we assume that this biopsy at the 2.5-year visit will not reveal cancer progression. The risk at year 3 is then calculated conditioning on progression not happening before this most recent (tentative) biopsy at $\tilde{t}^{(b)} = 2.5$. Note that we do not make any assumptions about the yet unknown values of the longitudinal outcomes between the current time and future visits, i.e., we use $\Pi_{i'}^{(\text{PRG})}\left\{t_7 \mid \tilde{t}^{(b)}, t^{(y)}\right\}$ where $t^{(y)}$ is still the same as before. Following this procedure, we plan biopsies at years $2.5, 5.5, 7.5$, and finally 10, as visualized in Figure S10.

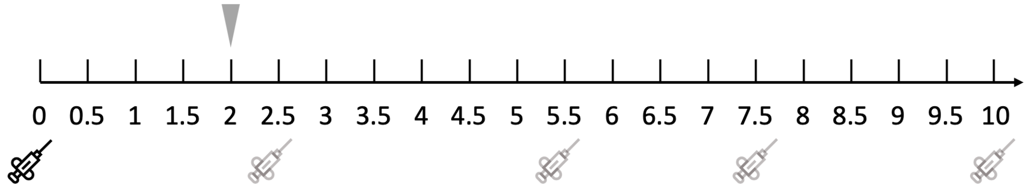

Figure S10: Patient i' at year 2.

Over time, when additional longitudinal measurements are taken, $t^{(y)}$ is updated as well. Consequently, the personalized schedules are re-generated each time $t^{(y)}$ is updated. Say a half year later, at $t_5 = 2.5$, a new PSA measurement was taken and the updated risk of progression at this time is 0.09 and is no longer above the risk threshold. Therefore, a biopsy is not conducted. However, at the following visit $t_6 = 3$, the again updated risk exceeds the boundary and, thus, a biopsy will be conducted. At this time, a new schedule is proposed, resulting in biopsies being scheduled at years $6, 8$, and 10 (Figure S11).

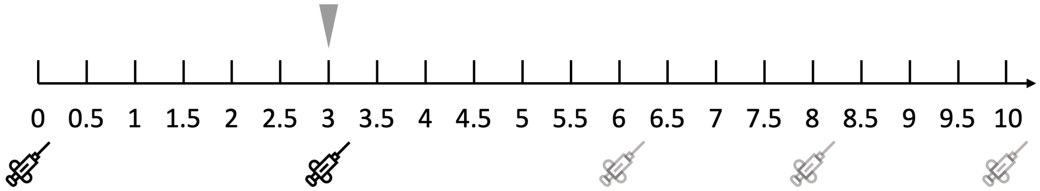

Figure S11: Patient i' at year 3.

References

[1] Tomer A, Nieboer D, Roobol MJ, Steyerberg EW, Rizopoulos D. Shared decision making of burdensome surveillance tests using personalized schedules and their burden and benefit. *Stat Med.* 2022;41(12):2115-2131.

[2] Plummer M. JAGS: A Program for Analysis of Bayesian Graphical Models using Gibbs Sampling. *3rd International Workshop on Distributed Statistical Computing (DSC 2003); Vienna, Austria.* 2003;124.

[3] R Core Team . *R: A Language and Environment for Statistical Computing.* R Foundation for Statistical Computing; Vienna, Austria: 2022.
